# A Narrative Review of Airway and Gut Microbiota in Chronic Obstructive Pulmonary Disease: Clinical Associations, Methodological Heterogeneity, and Translational Priorities

**DOI:** 10.7759/cureus.115698

**Published:** 2026-09-03

**Authors:** Ahmet Akcan

**Affiliations:** 1 Pulmonary Medicine, Afyonkarahisar Devlet Hastanesi, Afyonkarahisar, TUR

**Keywords:** airway microbiome, antibiotics, chronic obstructive pulmonary disease, dysbiosis, gut-lung axis, gut microbiota, inhaled corticosteroids, microbial load

## Abstract

Chronic obstructive pulmonary disease (COPD) is a heterogeneous disorder in which exacerbation susceptibility, persistent inflammation, disease progression, and treatment response are not fully explained by spirometry. Culture-independent studies have associated airway and gut microbial features with clinically relevant COPD phenotypes, but findings are highly sensitive to sampling site, low biomass, contamination control, sequencing depth and platform, bioinformatic workflow, microbial-load quantification, medication exposure, disease state, and host or environmental confounding. Methodological and clinical heterogeneity is therefore a central explanation for inconsistent results. This narrative review, a non-systematic synthesis using a prespecified focused PubMed/MEDLINE search (1 August 2021-1 August 2026), English-language eligibility, single-reviewer selection, structured data charting, and thematic appraisal without formal study-level risk-of-bias grading, evaluates recent human evidence on the airway bacteriome and mycobiome, gut microbiota and metabolites, host-microbe relationships, and the ecological effects of antibiotics and inhaled corticosteroids. Across cohorts, lower airway diversity, states dominated by potential pathobionts (normally resident organisms that may contribute to disease under altered host or ecological conditions), and altered microbial networks are recurrent but not universal associations; no disease-specific taxonomic signature has been validated. Gut microbial and metabolic differences may represent causes, consequences, treatment effects, shared determinants, or combinations of these mechanisms. Relative abundance is difficult to interpret without absolute microbial-load measurement. No microbiota-based diagnostic test, prognostic classifier, or intervention is ready for routine COPD care. Progress requires standardized longitudinal sampling, rigorous controls, absolute quantification, paired airway-gut multi-omics, diverse external validation, and randomized trials with prespecified patient-centered outcomes.

## Introduction and background

Chronic obstructive pulmonary disease (COPD) is a heterogeneous clinical condition arising from interactions between harmful environmental exposures and individual susceptibility. Persistent abnormalities of the airways and/or alveoli cause respiratory symptoms and incompletely reversible airflow limitation [1]. Spirometry establishes airflow obstruction but does not fully explain clinically important variation in inflammatory phenotype, symptom burden, exacerbation frequency and severity, hospitalization risk, rate of lung-function decline, mortality, or response to antibiotics and anti-inflammatory treatment [1]. Microbial ecology is relevant to this heterogeneity because differences in community structure, absolute microbial burden, intermicrobial interactions, functional potential, and host response may accompany distinct inflammatory and exacerbation-prone states [2-4]. These associations may eventually refine biological phenotyping, but they do not yet justify microbiome-guided treatment and should not be interpreted as proof of causality [1].

Microorganisms were historically considered in COPD mainly as causes of acute infection or as organisms persistently present in the airways without necessarily causing invasive disease. Culture-independent sequencing, which detects microbial genetic material without requiring growth in culture, has broadened this view by identifying diverse but low-biomass microbial communities in the lower respiratory tract. Research has consequently shifted from asking only whether a pathogen is present to examining community composition, diversity, stability, total microbial burden, intermicrobial relationships, and functional potential. Dysbiosis is used here as an ecological description of a disturbed microbial community or microbial function associated with an adverse host state. It is not synonymous with infection, and its operational definition remains inconsistent across COPD studies [2-4].

Foundational bronchoscopy and resected-lung studies overturned the assumption that healthy lower airways are sterile. They demonstrated low-biomass bacterial communities shaped by immigration from the upper airway, mucociliary clearance, local growth conditions, and host defense. COPD cohorts showed enrichment of *Proteobacteria*, including *Haemophilus*, and differences in community composition across disease severity and anatomical regions; however, low biomass and small samples made contamination controls and replication essential [2-4].

A commonly proposed model is a self-reinforcing cycle in which COPD-related airway obstruction, impaired mucociliary clearance, structural damage, hypoxia, altered mucus, and weakened mucosal defenses create conditions that may reshape airway ecology. Selected microbial configurations could, in turn, amplify epithelial injury or immune activation. This disease-driven pathway is biologically plausible but has not been established as a causal sequence in humans. It must be distinguished from treatment-associated dysbiosis. Antibiotics can directly reduce susceptible organisms, permit the expansion of resistant or previously suppressed taxa, alter total microbial load, and select antimicrobial-resistance genes. Inhaled corticosteroids (ICS) may modify local immune defense, bacterial abundance, and fungal ecology; systemic corticosteroids may have additional airway and systemic effects. Treatment-associated changes may be transient after a short course or accumulate with repeated and long-term exposure. Because antibiotics and ICS are preferentially given to patients with infection risk, frequent exacerbations, greater severity, or particular inflammatory phenotypes, an observed post-treatment profile may reflect the drug, the clinical indication, the underlying COPD state, or all three. Cross-sectional associations cannot reliably separate these pathways [4-8].

Microbiota research also extends beyond the airways. The gut microbiota can generate short-chain fatty acids, bile-acid derivatives, tryptophan metabolites, and other products that may influence epithelial barriers, innate immunity, T-cell responses, and systemic inflammation. Conversely, pulmonary inflammation, hypoxemia, reduced physical activity, altered diet, smoking, hospitalization, and medication use may reshape the intestinal ecosystem. This proposed bidirectional communication is termed the gut-lung axis. For non-specialist readers, three evidential levels should be kept distinct: an association means that two features occur together; biological plausibility means that an experimentally credible mechanism could connect them; and causality requires evidence that changing one feature changes the other through the proposed pathway. Current COPD studies often support association and plausibility but rarely establish human causality because shared exposures, disease severity, and treatment-related confounding can explain part or all of the relationship [9-13].

**Figure 1:**
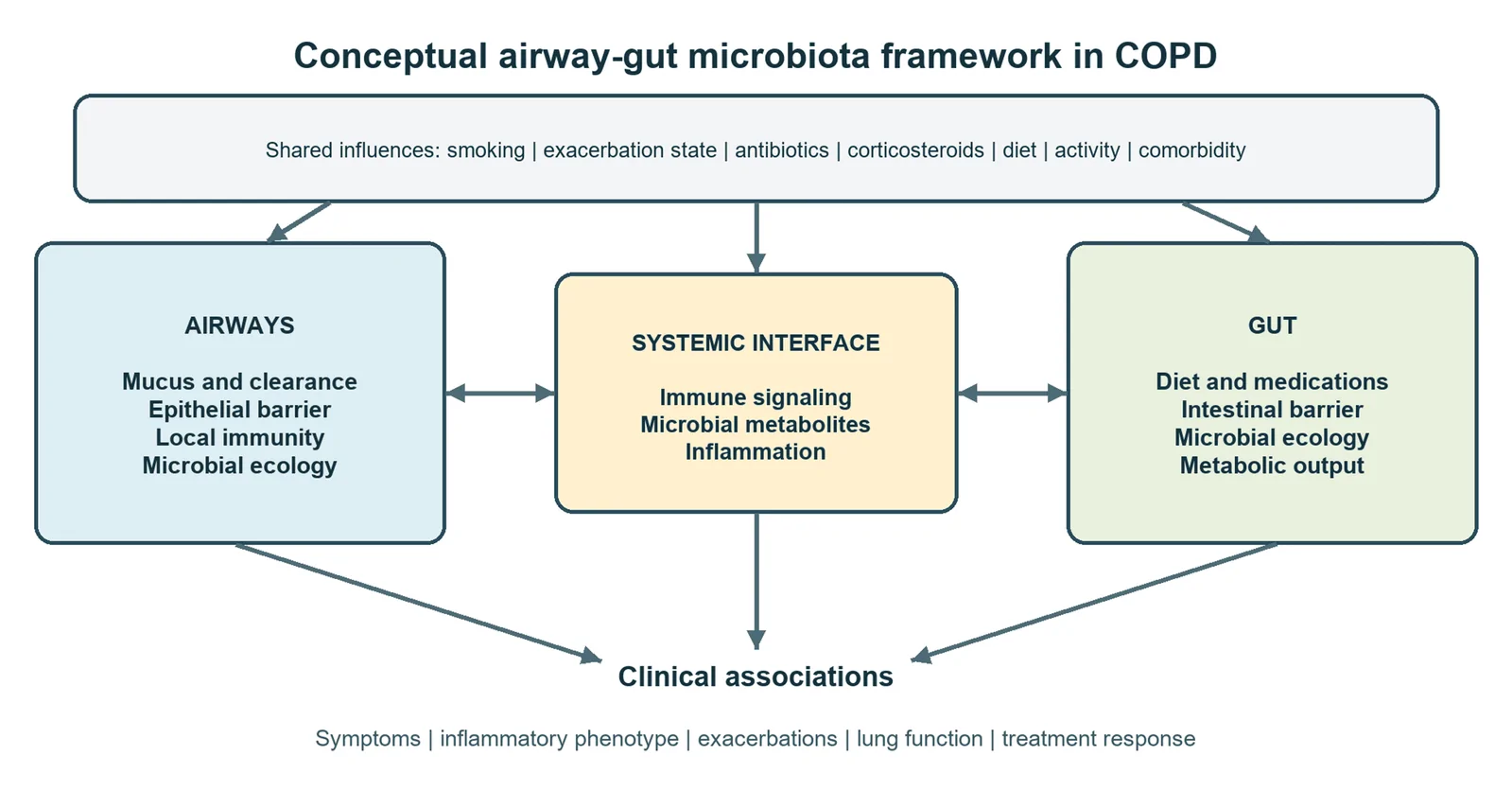
Conceptual airway-gut microbiota framework in COPD. Bidirectional immune and metabolic signaling is biologically plausible, but shared host, behavioral, disease-state, and treatment factors can influence both compartments and the observed clinical associations. COPD: chronic obstructive pulmonary disease

The Global Initiative for Chronic Obstructive Lung Disease (GOLD) 2026 recognizes airway dysbiosis and the gut-lung axis as evolving areas of COPD biology. Microbiome profiles may change during viral infection, an acute COPD exacerbation, antibiotic treatment, or exposure to ICS or systemic corticosteroids [1]. These contexts are not interchangeable: an exacerbation-related change may reflect the acute disease process, whereas a treatment-related change may result from antimicrobial selection or altered immune defense, and both may occur simultaneously. Limited longitudinal pre-treatment and post-treatment sampling, together with sparse interventional evidence, prevents reliable attribution of microbial changes to COPD rather than its treatment and prevents confident conclusions about causal direction, diagnosis, prognosis, or therapeutic utility. The objective of this review is therefore to evaluate four predefined dimensions of recent human evidence: (1) associations of airway bacterial and fungal communities with COPD phenotype, spirometric and clinical severity, inflammatory endotype, symptoms, exacerbations, hospitalization, lung-function trajectory, and mortality; (2) associations of gut microbiota and microbial metabolites with pulmonary and systemic outcomes; (3) ecological and host-response effects of antibiotics and ICS, including confounding by indication; and (4) methodological validity, cross-cohort reproducibility, biomarker performance, and readiness for clinical translation [1,8,14]. This bounded objective distinguishes description, prediction, biological plausibility, and causal evidence. Figure 1 summarizes the proposed bidirectional airway-gut framework and the shared influences that complicate causal interpretation.

Review methodology and evidence synthesis

Review Design

This focused narrative review synthesizes recent clinical and translational evidence; it was not prospectively registered or designed as a systematic review or meta-analysis and should not be interpreted as an exhaustive review. PubMed/MEDLINE was the only database searched systematically. The search and selection methods are reported to maximize transparency within this design, but the review is only partially reproducible: only English-language full texts were eligible; screening, data charting, and appraisal were conducted by one reviewer; exact study-flow counts and record-level exclusion reasons were not prospectively archived; and no validated study-level risk-of-bias instrument was applied. Consequently, no Preferred Reporting Items for Systematic Reviews and Meta-Analyses (PRISMA) flow diagram, pooled estimate, meta-regression, formal risk-of-bias summary, or certainty-of-evidence assessment was produced. PRISMA 2020 is not presented as a mandatory framework for this narrative review; relevant PRISMA principles and PRISMA-S concepts were used only to improve the reporting of information sources, search syntax, limits, and selection procedures. These design choices limit comprehensiveness and increase the possibility of selection and interpretation bias.

Eligibility Criteria

Reports were eligible if published from 1 August 2021 through 1 August 2026 and enrolled adults with COPD defined by GOLD criteria or an explicit clinical and spirometric definition and examined airway or gut microbial composition, microbial load, functional potential, metabolites, host response, clinical phenotype, disease severity, exacerbations, longitudinal outcomes, or treatment exposure. Observational, longitudinal, and interventional human studies using culture-independent analysis of sputum, induced sputum, bronchoalveolar lavage (BAL), bronchial samples, or feces were considered. Studies without separately reported COPD results, culture-only investigations, case reports, conference abstracts, editorials, and opinion articles were excluded from the recent primary-evidence synthesis. Only English-language full-text reports were eligible. This pragmatic restriction may have excluded relevant evidence, disproportionately omitted studies from some regions, and introduced language and publication bias; non-English records were not formally assessed for eligibility or effect on conclusions. Reviews were used for citation tracking and contextual interpretation rather than as primary evidence. Seminal studies published before the prespecified five-year window were cited only to establish historical, biological, or methodological context and were not treated as eligible recent studies when characterizing the contemporary evidence base.

Information Sources, Search Strategy, Study Selection, and Synthesis

PubMed/MEDLINE was the only bibliographic database searched systematically for records published from 1 August 2021 through 1 August 2026; the search was supplemented by the current GOLD strategy report and backward citation searching of eligible studies and relevant reviews. The final search covered the prespecified window ending 1 August 2026. The exact PubMed syntax was as follows: (("Pulmonary Disease, Chronic Obstructive"[Mesh] OR COPD[Title/Abstract] OR "chronic obstructive pulmonary disease"[Title/Abstract]) AND ("Microbiota"[Mesh] OR microbiome[Title/Abstract] OR microbiota[Title/Abstract] OR dysbiosis[Title/Abstract] OR metagenom*[Title/Abstract] OR bacteriome[Title/Abstract] OR mycobiome[Title/Abstract] OR virome[Title/Abstract] OR "gut-lung axis"[Title/Abstract] OR "gut lung axis"[Title/Abstract] OR airway[Title/Abstract] OR sputum[Title/Abstract] OR "bronchoalveolar lavage"[Title/Abstract] OR stool[Title/Abstract] OR fecal[Title/Abstract] OR metabolom*[Title/Abstract] OR "short-chain fatty acid*"[Title/Abstract])) AND ("2021/08/01"[Date - Publication]:"2026/08/01"[Date - Publication]) AND English[Language]. Human eligibility was assessed during screening rather than imposed as a filter because newly indexed records may lack complete indexing. No searches were conducted in Embase, Scopus, Web of Science, CINAHL, or dedicated preprint and grey-literature sources. The evidence retrieval should therefore be regarded as focused rather than comprehensive, particularly for multidisciplinary metabolomic, bioinformatic, and conference-indexed research. Exact search-result, deduplication, full-text exclusion, and inclusion counts were not prospectively archived; reconstructing these values retrospectively would be unreliable and was not attempted. The specimen and anatomical terms (airway, sputum, BAL, stool, and fecal) were intentionally included in the same broad concept block to maximize sensitivity for inconsistently indexed studies. This choice reduced search specificity and probably retrieved many non-microbiome COPD records, thereby increasing the amount of judgment required during single-reviewer screening. The strategy was not formally validated against a prespecified sentinel set of known eligible studies, and no independent information specialist peer review of the search was performed.

One author screened titles and abstracts against the stated population, specimen, method, outcome, publication-type, date, and language criteria, then assessed available full texts, charted study information, and performed the qualitative appraisal. Records encountered more than once through PubMed or citation searching were treated as one study when DOI, PMID, or title-author-year information matched. Reference lists were searched manually. Reasons for exclusion were applied at full text but were not prospectively stored as an auditable record-level log. Duplicate independent screening, duplicate data charting, and adjudication were not performed. The final study set and the relative emphasis assigned to individual findings therefore depended on the judgment of a single reviewer, creating a material risk of selection, confirmation, and interpretation bias that should be considered when evaluating the conclusions.

Data Charting and Thematic Synthesis

For each eligible recent primary study, information was charted narratively for author and year, design, population and sample size, clinical state or treatment exposure, anatomical sampling site, laboratory and sequencing approach, principal microbiome or host-response finding, clinical endpoint, confounder adjustment, longitudinal follow-up, external validation, and major methodological or inferential limitation. Selected seminal or context-setting primary studies published before the prespecified window were charted separately for historical or methodological interpretation. A purposively selected subset of recent and context-setting studies rather than a complete inventory is presented in a table. Studies were prioritized when they contributed at least one prespecified feature: a comparatively large or well-phenotyped cohort; longitudinal, paired, or randomized sampling; external or multicohort validation; direct relevance to patient-centered outcomes; inclusion of microbial-load, multi-omic, host-response, or treatment-exposure data; geographic or specimen diversity; or substantial methodological influence. Inclusion does not imply low risk of bias or greater certainty than studies not tabulated. Because a complete included-study inventory and archived extraction file are not available, readers can trace the studies discussed in the text and in the table to the reference list but cannot audit an exhaustive study universe or verify that every eligible record received equivalent evidential weight. This limitation should be distinguished from the purposeful selection criteria used for the representative table.

Studies were grouped a priori into airway bacteriome and community structure; exacerbations and host inflammation; mycobiome; gut microbiota and gut-lung mechanisms; treatment exposures and microbiota-directed interventions; and methodological or translational evidence. Greater interpretive weight was given to larger or longitudinal studies, paired or repeated sampling, direct microbial-load measurement, adjustment for major confounders, independent validation, and patient-centered outcomes. Convergence was assessed separately for ecological features and individual taxa.

Statistical Synthesis and Reporting

No meta-analysis, meta-regression, or formal heterogeneity statistic was undertaken because the included evidence differed substantially in populations, clinical states, specimens, platforms, taxonomic resolution, exposure definitions, covariate models, and outcomes. The synthesis was descriptive and thematic. Study-specific sample sizes, effect estimates, 95% confidence intervals, and p-values were reproduced from source reports when they described a principal clinical outcome, quantified the direction or magnitude of a central association, conveyed estimate precision or uncertainty, or materially clarified a null or subgroup finding; no values were recalculated or pooled. Numerical results were not selected solely because they reached statistical significance. Statistical significance was not treated as evidence of causality, absence of significance was not interpreted as equivalence, and vote counting was not used. Interpretation prioritized magnitude, precision, study design, confounder control, longitudinal or interventional evidence, external validation, and clinical relevance.

Evidence Appraisal and Reproducibility Statement

No validated design-specific risk-of-bias instrument, standardized certainty framework, or duplicate data extraction was used. Instead, the review applied a structured narrative appraisal across participant selection, case-control comparability, sample size, sampling site and timing, contamination controls, microbial biomass, sequencing depth and bioinformatics, missing data, medication exposure, confounder measurement and adjustment, outcome ascertainment, longitudinal follow-up, selective reporting, multiplicity, and external validation. Each study's design-based evidence category and principal methodological or inferential limitation are identified to make differences in evidential strength more visible. These descriptors are not validated risk-of-bias ratings and should not be interpreted as low, moderate, or high certainty labels. Another investigator can reproduce the stated PubMed query, date and language limits, and eligibility framework, but cannot exactly reproduce the final study set, exclusions, data charting, or weighting decisions because a protocol, complete search export, record-level screening log, duplicate review, and formal appraisal file were not retained. The review is therefore partially reproducible, not fully reproducible.

## Review

Airway microbiota

COPD Heterogeneity and Community Structure

Recent studies do not support a universal or disease-specific taxonomic signature for the COPD airway microbiome. Alpha diversity describes richness and evenness within an individual sample, whereas beta diversity describes differences in community composition between samples or groups. Selected case-control and longitudinal cohorts have reported lower alpha diversity, greater dominance by potential pathobionts, and altered microbial load or predicted functional potential, especially in exacerbation-prone or clinically severe subgroups; however, direction and magnitude vary by sampling site, clinical state, treatment exposure, geography, and analytical pipeline. A shift from diverse, commensal-rich communities toward less diverse or pathobiont-dominated states is therefore better described as a potentially reproducible ecological feature in particular phenotypes than as a defining COPD signature [2-4,14,15].

In an untargeted metagenomic study of 39 patients with stable COPD, Li et al. observed lower Shannon and Simpson diversity in frequent than in non-frequent exacerbators; alpha diversity was inversely associated with exacerbation frequency but not significantly associated with percent-predicted forced expiratory volume in one second (FEV1) [16]. The finding is hypothesis-generating because the study was small, cross-sectional, and based on one sampling time point. It neither establishes that reduced diversity precedes exacerbations nor demonstrates prognostic performance or clinical utility. Disease severity, smoking, prior exacerbations, antibiotics, ICS, and other host or treatment factors could partly or wholly account for the association; larger longitudinal and externally validated studies are required.

Community classification and network analyses further illustrate disease heterogeneity and may be more informative than isolated taxon abundance. Si et al. identified sputum metacommunities that differed in pathobiont abundance, inferred functional potential, and microbial interaction structure [17]. In a pooled analysis of 1,742 sputum microbiomes, negative bacterial associations were less frequent in COPD than in healthy controls. Alteration of the antagonistic association network surrounding *Haemophilus*, rather than *Haemophilus* abundance alone, was described as a potentially reproducible ecological feature [18]. These networks are statistical inferences from compositional data, not direct demonstrations of biological interaction; cohort heterogeneity and unmeasured confounding further limit causal interpretation, and prospective validation is required before clinical use.

Larger cohorts have refined this ecological model while also illustrating how study design and specimen type shape inference. In the bronchoscopic SubPopulations and InteRmediate Outcome Measures in COPD Study (SPIROMICS) analysis, protected lower-airway sampling reduced but did not eliminate upper-airway contamination and linked bacterial community variation with bronchodilator responsiveness, small-airway flow, symptoms, and functional capacity [15]. However, bronchoscopy is invasive, participation was restricted to a smaller, selected mild-to-moderate subgroup, and the cross-sectional associations may not generalize to patients with very severe disease or acute exacerbations. A subsequent sputum analysis of 877 SPIROMICS participants increased sample size and enabled longitudinal clinical assessment; lower phylogenetic diversity was associated with worse lung function, symptoms, radiographic small-airway disease, mucin concentrations, and faster lung-function decline [14]. Sputum nevertheless represents a mixed upper- and lower-airway specimen and is sensitive to oral admixture, expectoration quality, microbial biomass, sequencing depth, and sample-processing decisions. Although multivariable models strengthen these analyses, adjustment does not eliminate residual confounding. Important potential confounders include current smoking status, pack-years, time since smoking cessation, ICS molecule and cumulative exposure, recent and cumulative antibiotic or systemic corticosteroid exposure, exacerbation history, baseline disease severity, oral health, comorbidities, socioeconomic conditions, environmental exposures, and sampling or treatment timing. SPIROMICS therefore provides important, well-phenotyped cohort evidence, but the reported relationships remain adjusted observational associations rather than proof that loss of diversity causes progression or that restoring diversity would improve outcomes.

A 2026 prospective cohort added longer-term temporal context by collecting 129 annual sputum samples from 43 Korean men with COPD over two years, with 10 male controls at baseline. ICS use, continued smoking, and lower FEV1 were associated with distinct taxonomic trajectories, whereas recent exacerbation was not significantly associated with composition in the fitted models [19]. Repeated sampling is a strength, but the small, all-male, geographically restricted cohort, annual sampling interval, sputum oral admixture, and time-varying treatment and smoking exposures limit generalizability and causal interpretation.

Exacerbations, Recovery, and Host Inflammation

Reduced bacterial diversity and relative enrichment of *Proteobacteria* are among the most frequently reported findings during acute exacerbations. In a study comparing acute exacerbation, stable disease, recovery, and healthy-control samples, diversity was lower in the exacerbation and recovery groups, and *Proteobacteria* was the dominant phylum during acute exacerbation [20]. Persistently reduced diversity during recovery may indicate that ecological recovery lags behind clinical improvement; alternatively, antibiotics and corticosteroids administered during the exacerbation may influence post-treatment samples.

Integrated host-microbiome analyses have sought to connect community changes with inflammatory pathways. A metagenomic and host RNA-sequencing study reported differences in *Rothia mucilaginosa* and *Haemophilus influenzae* between acute exacerbation and stable COPD and linked host transcriptional changes to type I interferon, cytosolic DNA-sensing, Toll-like receptor, and tumor necrosis factor pathways [21]. Such multi-omic designs offer mechanistic insight but are vulnerable to false-positive and cohort-specific findings because of small samples, high-dimensional testing, and limited independent validation.

Inflammatory endotyping illustrates why one taxonomic signature is unlikely to represent all COPD. A multicohort analysis of 1,706 longitudinal sputum samples identified a relatively stable *Haemophilus*-predominant neutrophilic state associated with interleukin-1 beta and tumor necrosis factor signaling and a more diverse, temporally unstable interleukin-17A-associated state that shifted more during exacerbations [8]. The Acute Exacerbation and Respiratory InfectionS in COPD (AERIS) study used repeated longitudinal sputum sampling and classified exacerbation events using microbiological and inflammatory features. Participants who experienced a bacterial-associated exacerbation, or an eosinophilic exacerbation, had a higher probability that a subsequent exacerbation would display the same respective biological category [7]. This finding indicates within-person tendency or phenotype repeatability at the group level; it does not mean that successive events in every participant shared an identical cause, that the categories were mutually exclusive, or that the associated airway microbiota caused the subsequent exacerbation phenotype. Antibiotic and corticosteroid exposure, sampling timing, and imperfect event classification may also affect observed repeatability.

A 2026 hospital-based analysis further examined short-term treatment response by blood-eosinophil phenotype. Among 202 acute exacerbation of chronic obstructive pulmonary disease (AECOPD) hospitalizations, clinical outcomes differed by an admission eosinophil threshold of 2%; in a prospective microbiome subcohort of only 30 patients, sputum diversity and *Proteobacteria* changed differently between days 1 and 5 [22]. Because treatment components were not experimentally isolated, the microbiome sample was small, and pneumonia and other clinical factors may confound comparisons, these findings support phenotype-specific hypotheses rather than a microbiome-guided treatment rule.

Overall, airway studies support dynamic, phenotype-dependent ecological states rather than a fixed COPD microbiome. This evidence includes well-phenotyped bronchoscopic cohorts, large sputum cohorts, repeated sampling, multicohort analyses, and limited randomized treatment comparisons; nevertheless, it remains predominantly associative. Exacerbation susceptibility, inflammatory phenotype, antibiotics, and corticosteroids may influence ecological transitions, which should be interpreted using diversity, absolute burden, network structure, functional potential, and longitudinal clinical context [5-8,14,15,21].

Airway Mycobiome: Detection, Colonization, Dysbiosis, Infection, and Fungal-Bacterial Interactions

Fungal presence must be separated explicitly from colonization, sensitization, dysbiosis, and clinically significant fungal disease. Detection of fungal nucleic acid, antigen, or culture growth may reflect transient inhalation, contamination, nonviable material, or viable organisms. Colonization denotes persistent or repeated airway recovery without tissue invasion or a fungus-attributable syndrome; sensitization requires a specific host immune response but does not itself establish infection. Dysbiosis is an ecological descriptor, whereas clinically significant fungal disease requires a compatible syndrome supported by radiological, immunological, microbiological, and, where appropriate, histopathological evidence [23]. Viability and clinical relevance are strengthened by reproducible culture, repeated longitudinal detection, RNA-based evidence of activity, organism-specific quantitative assays, fungal biomarkers such as galactomannan when clinically appropriate, and concordant fungus-specific immune findings; no single test is sufficient in every setting. In a multicenter study including 337 stable COPD participants, 66 exacerbation samples, and 47 controls, a cluster containing *Aspergillus*, *Curvularia*, and *Penicillium* was associated with very frequent exacerbations, fungal sensitization, and mortality, while lower fungal diversity during exacerbation was associated with two-year mortality [24]. These findings identify a candidate risk marker, not a causal pathway. Reverse causation and confounding by COPD severity, structural lung disease, antibiotics, ICS or systemic corticosteroids, comorbidity, and healthcare exposure remain plausible. Fungal-bacterial interactions may involve nutrient competition, biofilm organization, cross-kingdom signaling, antimicrobial exposure, and host immunity, but COPD-specific evidence remains limited.

Gut microbiota and the gut-lung axis

Gut Microbial Signatures and Clinical Phenotypes

Case-control studies have reported differences in gut microbial composition and inferred functional potential between patients with COPD and controls, but these findings do not constitute a consistent, disease-specific, or causal microbiome signature. Both the direction and magnitude of alpha-diversity and taxonomic differences vary among cohorts. Age, sex, body mass index, diet, geography, socioeconomic conditions, smoking status and pack-years, physical activity, oral health, alcohol use, comorbidities, proton-pump inhibitors, antibiotics, ICS or systemic corticosteroids, other medications, and COPD severity can differ substantially between COPD groups and controls. Inadequate matching, measurement, or adjustment for these variables can generate apparent disease-associated differences, attenuate genuine associations, or reverse their direction [9-13]. COPD-control comparisons should therefore be interpreted as context-dependent observational associations, with explicit consideration of selection bias, residual confounding, batch effects, and multiple testing, rather than evidence of a universal COPD microbiome.

In a metagenomic case-control study, Li et al. compared 80 patients with COPD and 80 healthy controls and reported stage-associated taxonomic and functional differences. Microbial classifiers discriminated COPD from control samples within the study dataset [9]. Such performance may be optimistic when model development and evaluation occur in closely related datasets. Claims of diagnostic utility require geographically diverse, external cohorts with robust adjustment for age, smoking, diet, proton-pump inhibitor use, antibiotics, and comorbidity.

Longitudinal evidence is more informative than cross-sectional classification but remains observational. A one-year study associated changes in gut microbial communities with lung-function decline in treated stable COPD [10]; limited sample size, short follow-up, diet, and medication exposure constrain causal inference. In FINRISK 2002, baseline fecal metagenomes from 7,115 population participants were linked to national health records for up to 15 years, and microbial features were associated with future chronic respiratory disease [11]. Its population scale, prospective linkage, and long follow-up make it valuable for hypothesis generation about respiratory risk. However, the outcome combined chronic respiratory diagnoses rather than rigorously phenotyping incident COPD, baseline spirometry was incomplete, and registry coding cannot fully distinguish COPD from asthma or other lung disease. FINRISK therefore supports a possible association between baseline gut microbial features and later respiratory morbidity, but it should not be presented as COPD-specific prediction or evidence that gut dysbiosis causes COPD.

Bowerman et al. combined shotgun metagenomics and untargeted metabolomics and identified disease-associated species and metabolites correlated with lung function [13]. This paired profiling offers a hypothesis-generating view of taxonomic and metabolic covariation, not an unqualified functional bridge between intestinal microbiota and COPD. Metagenomic pathways indicate encoded functional potential, and measured metabolites may derive from microbial, host, dietary, or drug metabolism; correlation between these layers does not identify source, flux, mediation, or causality. Cross-sectional findings may also reflect age, body mass index, diet, smoking, medication, comorbidity, body composition, or reduced activity. Later studies extend these associations across cohorts [9-12], but current human evidence cannot determine whether gut dysbiosis is a cause of COPD, a consequence of pulmonary and systemic disease, a treatment-induced effect, a product of shared confounders, or a mixture of these processes.

Mechanistic Interpretation

The gut-lung axis is a biologically plausible framework, but established general biology must be separated from COPD-specific evidence. Across experimental systems and other disease contexts, short-chain fatty acids, tryptophan derivatives, bile acids, lipopolysaccharides, epithelial barriers, macrophages, and T-cell responses can participate in interorgan immune and metabolic signaling. Recent COPD-focused reviews published in 2026 synthesize these pathways and proposed microbiota-directed strategies, but their mechanistic and therapeutic conclusions rely substantially on preclinical studies and heterogeneous observational evidence [25]. In COPD, direct human evidence that a defined gut microbial exposure changes these mediators and thereby alters airway inflammation, exacerbations, lung-function decline, or mortality remains limited. COPD-related inactivity, dietary change, hypoxemia, smoking, systemic inflammation, age, body mass index, comorbidity, and recurrent medication exposure may independently affect intestinal ecology and circulating metabolites. Biological plausibility should therefore be distinguished from directly measured mediation, COPD-specific mechanism, and established causality [9-13].

A recent prospective, multicenter, controlled study jointly evaluated oropharyngeal swabs, sputum, BAL, and fecal samples from patients with stable COPD and controls. Microbial similarity across anatomical compartments within individuals was low, whereas selected microbial modules were associated with FEV1, dyspnea, exacerbation history, and ICS use [12]. This supports the conceptual importance of functional and metabolic crosstalk over simple taxonomic overlap, but it does not directly demonstrate microbial activity or gut-to-lung transfer. Here, microbial modules represent co-occurrence or inferred functional patterns; confirmation requires measured metabolites, transcripts, proteins, isotope tracing, or other activity and mediation assays together with longitudinal clinical data.

A 2025 paired-sample study evaluated 24 patients before and after the treatment of an acute COPD exacerbation and reported post-treatment changes in gut and pharyngeal richness and composition, while fecal short-chain fatty acids and most measured circulating inflammatory mediators did not change [26]. The within-person design is informative for treatment-associated perturbation, but the small sample, combined treatment exposure, absence of an untreated comparator, pharyngeal rather than lower-airway sampling, and multiple taxonomic comparisons prevent attribution to COPD recovery, antibiotics, corticosteroids, or another treatment component.

Integrated metagenomic and metabolomic analyses can identify associations among genes, predicted pathways, metabolites, and clinical traits, but the phrase microbial function should be reserved for directly demonstrated activity. Amplicon-based prediction and DNA-based pathway reconstruction represent inferred or encoded functional potential, not function itself. Metatranscriptomics, metaproteomics, metabolomics, stable-isotope tracing, culture-based assays, and validated biochemical measurements provide progressively more direct but still context-dependent evidence; even a metabolite cannot automatically be assigned a microbial origin. Throughout this review, reports based only on gene content or computational reconstruction are therefore described as predicted or inferred functional potential. Candidate signals from high-dimensional datasets remain vulnerable to overfitting, multiple-testing error, database annotation limitations, batch effects, and cohort-specific dietary influences. Translation requires prespecified targets, targeted assays, independent replication, and evidence of incremental value beyond standard clinical predictors [13,27].

Virome evidence is emerging and should not be conflated with routine testing for acute respiratory viral pathogens. The virome includes eukaryotic viruses and bacteriophages that may influence bacterial ecology, horizontal gene transfer, antimicrobial resistance, and host immunity. A Chinese case-control study of 92 participants reported fecal virus-like particle differences, phage-bacterial correlations, and an internally evaluated COPD classifier [28]. Because the study assessed fecal virus-like particles, it informs gut virome ecology rather than active respiratory infection. Cross-sectional design, geographic restriction, incomplete reference databases, uncertain phage-host assignment, and lack of external validation preclude diagnostic or causal claims. Longitudinal paired airway-stool studies with quantitative viral load and evidence of activity are required to determine whether virome changes precede clinical events or reflect disease and treatment.

Host-response measurements can help distinguish community changes associated with active host responses from passive detection. Bronchial brushings from a nine-center European cohort linked COPD-associated bacterial patterns with epithelial transcriptional programs [29]. Lin et al. integrated BAL metagenomics and host transcriptomics across discovery and validation cohorts and used quantitative polymerase chain reaction (qPCR) to validate selected microbial-immune markers associated with COPD severity [30]. The BAL design and independent validation strengthen internal credibility, but cross-sectional sampling cannot establish temporal direction. Disease severity, chronic treatment pressure, antibiotic or ICS exposure, and residual confounding may also contribute to the reported microbial, immune, and resistance-gene associations. These data support mechanistic hypotheses rather than immediate precision-treatment claims.

Treatments and microbial ecology

Antibiotics and Corticosteroids

Medication exposure is potentially one of the largest sources of confounding in observational COPD microbiome studies. This ecological discussion does not question guideline-concordant antibiotic treatment when a bacterial exacerbation is clinically suspected or confirmed; it addresses how treatment changes microbial measurements and complicates causal interpretation. Antibiotics, ICS, systemic corticosteroids, proton-pump inhibitors, maintenance macrolides, and other therapies may modify community structure, biomass, fungal ecology, metabolites, host defense, and resistance genes. Because these drugs are preferentially prescribed to patients with infection, greater severity, frequent exacerbations, or specific inflammatory phenotypes, observed profiles may reflect both direct effects and confounding by indication. Acute courses should be separated from recent and cumulative long-term exposure. Studies should report class, formulation, dose, route, duration, adherence, indication, co-treatment, cumulative exposure, and sampling time. Long-term macrolides reduce exacerbations in selected patients, but resistance and other adverse effects remain relevant; microbiome modification should not be assumed to be the sole mechanism [1,5-7,31]. The airway and gut resistome should be evaluated alongside taxonomic, load, metabolite, safety, and patient-centered outcomes.

ICS exposure may influence several biologically distinct components of airway ecology: bacterial community composition, total bacterial abundance, fungal community structure, microbial clearance, epithelial barrier and antimicrobial responses, macrophage and neutrophil function, cytokine signaling, and other local innate or adaptive immune mechanisms. An increase in bacterial or fungal detection should not automatically be interpreted as clinical infection, and changes in relative abundance should be separated from changes in absolute microbial load. Confounding by indication is a central threat to observational ICS-microbiome inference, not a minor statistical caveat. ICS is not allocated randomly in routine care: patients receiving it, particularly at higher doses or for longer periods, commonly differ from untreated patients in exacerbation history, airflow limitation, eosinophilic phenotype, prior infection, pneumonia risk, healthcare contact, adherence, and concurrent therapy. Each of these factors may independently affect microbial load, bacterial and fungal profiles, host immunity, and the probability or timing of sampling. Adjustment for measured covariates cannot reliably remove unmeasured severity, clinician treatment-selection processes, reverse causation, or time-varying confounding; an apparent ICS-associated microbial pattern may therefore predate treatment or reflect the indication for treatment. Stronger designs should use new-user or active-comparator approaches, repeated pre-treatment sampling, time-updated exposure models, propensity or weighting methods where appropriate, and randomized evidence. Studies should also separate initiation or short exposure from sustained maintenance and cumulative long-term exposure and report molecule, formulation, device, dose, duration, adherence, indication, recent systemic corticosteroid use, co-medication, and sampling time. Even randomized comparisons remain limited by attrition, selected eligibility, concurrent bronchodilator therapy, withdrawal or carryover effects, short follow-up, and incomplete characterization of microbial load, fungal ecology, host responses, and the resistome [32,33].

Randomized evidence suggests that ICS-containing regimens can influence airway ecology, but DISARM and MUSIC do not establish that fluticasone is intrinsically or universally worse for the microbiome. In DISARM, fluticasone/salmeterol was associated with more pronounced longitudinal community shifts than budesonide/formoterol or formoterol alone [32]. Nominal doses of different ICS molecules are not necessarily pharmacologically or biologically equivalent; glucocorticoid potency, lipophilicity, residence time, formulation, particle size, inhaler device, lung deposition, adherence, and bronchodilator partner may all influence exposure. The trial therefore cannot isolate a molecule-specific effect. In MUSIC, 122 participants entered a four-week ICS-withdrawal run-in; 61 withdrew before randomization, including 45 who experienced an exacerbation, and the remaining 61 participants were randomized to one of four inhaled regimens for three months. The trial therefore evaluated regimen-specific effects after an ICS washout in a selected population, not treatment initiation in ICS-naive patients or long-term effects in an unselected COPD population. High-dose fluticasone/salmeterol increased sputum bacterial load relative to budesonide/formoterol without a clear between-group alpha-diversity difference [33]. Withdrawal-related attrition, selected eligibility, modest sample size, baseline variation, carryover, and chance constrain inference. These findings are regimen-specific and hypothesis-generating.

Long-term macrolide treatment also creates an ecological trade-off. Clinical trials demonstrate reduced exacerbation rates in selected frequent exacerbators, whereas genomic surveillance during azithromycin exposure has documented within-host adaptation and acquisition of macrolide resistance in persistently colonizing *Haemophilus* species [31]. Consequently, the clinical benefit of long-term macrolide therapy must be weighed against the selection for antimicrobial resistance. Routine sequencing cannot presently identify which individual patient will obtain a favorable benefit-risk balance.

Microbiota-Directed Interventions

Microbiota-directed interventions are not interchangeable. Probiotics, prebiotics, synbiotics, regulated live biotherapeutic products, targeted microbial or metabolite interventions, and fecal microbiota transplantation (FMT) differ in composition, mechanism, manufacturing, regulation, and risk. FMT additionally requires rigorous donor screening and surveillance for pathogen transmission, antimicrobial-resistance genes, unintended metabolic or immune effects, and adverse outcomes in vulnerable or immunocompromised recipients. Live products require strain-level identity, purity, potency, stability, absence of clinically important resistance or virulence determinants, and manufacturing consistency. COPD evidence is not equally negative or equally sparse across categories. A small pilot randomized study tested oral *Lactobacillus rhamnosus *GG, inhaled amikacin, or vaccination without microbiome profiling, preventing treatment-specific mechanistic conclusions [34]. A 2025 exploratory study compared 32 patients with 32 controls and randomized 32 patients to a multi-nutrient product containing prebiotic fibers or placebo; no significant microbiome or systemic-inflammatory effect was detected [35]. With only 16 participants per arm and several active co-ingredients, the result neither isolates a prebiotic effect nor excludes effects of other formulations, doses, durations, or endpoints.

Future intervention studies should report intervention class, strain or product identity, dose, viability, manufacturing quality, co-ingredients, duration, adherence, safety, and predefined stopping rules. Safety assessment should include infection, aspiration, or procedure-related harm where relevant, gastrointestinal events, immune complications, and transmission or enrichment of resistance and virulence determinants. A primary patient-centered outcome should be prespecified; microbiome, metabolite, inflammatory, and resistome changes indicate biological engagement but are not validated surrogates for clinical benefit. Evidence remains insufficient for routine use, and its maturity differs substantially across intervention categories.

The characteristics and principal findings of representative studies are summarized in Table 1. Interpretation of these studies is also informed by low-biomass contamination guidance [36,37] and by geographically and phenotypically informative cohorts used to evaluate cross-cohort reproducibility [38,39].

**Table 1:** Characteristics, evidential position, and principal limitations of representative human studies informing the review. Evidence categories describe design and inferential position rather than certainty: cross-sectional association identifies contemporaneous covariation; longitudinal association establishes temporal observation without necessarily establishing causation; prognostic association evaluates a future clinical outcome; mechanistic plausibility links observations to a credible pathway without proving mediation; treatment-associated ecological change describes change during or after an exposure; randomized comparative evidence reflects allocated treatment comparison; and clinical-utility evidence requires demonstration that using a marker or intervention improves patient outcomes. These categories are not formal certainty or risk-of-bias grades. Studies published before the prespecified five-year window are included only when they provide essential historical, methodological, or context-setting evidence. The final column highlights the principal limitation and does not replace full appraisal of biomass, negative and positive controls, spike-in procedures, sequencing depth, absolute versus relative abundance, extraction and analytical batch effects, medication timing, confounder adjustment, and external validation in the source report. Reporting of these domains was inconsistent and was not inferred when absent. 16S: 16S ribosomal RNA gene sequencing; AECOPD: acute exacerbation of chronic obstructive pulmonary disease; BAL: bronchoalveolar lavage; COPD: chronic obstructive pulmonary disease; ICS: inhaled corticosteroid; ITS: internal transcribed spacer sequencing; LABA: long-acting beta2-agonist; qPCR: quantitative polymerase chain reaction; RNA-seq: RNA sequencing

Study	Design, specimen, and evidence category	Clinical state/exposure	Principal association or finding	Structured critical appraisal: principal methodological or inferential limitation
Li et al., 2022 [16]	Cross-sectional observational; case-control; stable COPD (n=39); sputum shotgun metagenomics	Frequent vs non-frequent exacerbators	Lower diversity associated with exacerbation frequency	Small, cross-sectional cohort; treatment and severity confounding; no causal inference
Si et al., 2022 [17]	Cross-sectional/community typing with external cohort; healthy cohort (n=202); external COPD cohort (n=324); sputum network analysis	Mixed respiratory states	Metacommunities differed in pathobionts and inferred interactions	Compositional network inference; heterogeneous cohorts
Su et al., 2022 [20]	Cross-sectional clinical-state comparison; AECOPD, stable, recovery, controls; 76 sputum samples; 16S	Before/after clinical state; treatment exposure relevant	Diversity remained lower during exacerbation and recovery	Treatment and disease-state effects not separable
Li et al., 2023 [9]	Cross-sectional case-control/classifier development; COPD (n=80); controls (n=80); stool shotgun metagenomics	Case-control	Taxonomic and pathway classifiers reported	Internal performance; external and geographic validation required
Xiao et al., 2024 [18]	Pooled multicohort observational network analysis; pooled 1,742 sputum microbiomes; interaction networks	Stable and exacerbation datasets	Reduced negative interactions; *Haemophilus* network signal	Cross-study platform and pipeline heterogeneity
Li et al., 2024 [21]	Cross-sectional multi-omic observational; AECOPD (n=31); stable COPD (n=26); sputum metagenomics/RNA-seq	Exacerbation vs stability	Microbial features associated with inflammatory pathways	Small, high-dimensional study; limited independent validation
Chiu et al., 2022 [10]	Longitudinal observational; one-year stable-COPD follow-up; stool 16S	Treated longitudinal cohort	Gut changes associated with lung-function decline	Diet and medication variability; limited duration and sample size
Viglino et al., 2026 [12]	Prospective multicenter controlled study; paired-compartment microbiome analysis; COPD (n=60); controls (n=30); oropharynx, sputum, BAL, and stool	Stable disease; ICS exposure assessed	Low cross-site similarity; modules associated with clinical traits	Cross-sectional associations; compartment-specific contamination risks
Wang et al., 2021 [8]	Multicohort longitudinal observational; 510 patients; 1,706 longitudinal sputum samples; 16S/mediators	Stability and exacerbations	Distinct neutrophilic ecological states	Phenotype associations do not establish microbial causality
Opron et al., 2021 [15]	Cross-sectional bronchoscopic cohort; SPIROMICS BAL (n=181); 16S	Mild-to-moderate COPD; bronchoscopic sampling	Community variation associated with clinical traits	Invasive sampling and selected subgroup limit generalizability
Opron et al., 2024 [14]	Large longitudinal observational cohort; SPIROMICS sputum (n=877); longitudinal 16S	Clinical and radiographic follow-up	Lower diversity associated with adverse traits and decline	Oral admixture and residual treatment/smoking confounding
Tiew et al., 2021 [24]	Multicenter observational mycobiome cohort; 337 stable COPD, 66 exacerbation samples, 47 controls; ITS	Stable/exacerbation; sensitization assessed	Fungal cluster associated with exacerbations and mortality	Association does not establish infection or causality; external validation needed
Bowerman et al., 2020 [13]	Cross-sectional multi-omic case-control; COPD case-control; stool metagenomics/metabolomics	Case-control	Species and metabolites correlated with lung function	Cross-sectional; functional and causal claims remain hypothesis-generating
Leitao Filho et al., 2021 [32]	Randomized longitudinal treatment trial; DISARM randomized trial; longitudinal sputum 16S	ICS/LABA formulations and formoterol	Regimen-dependent community shifts	Small trial; formulation, dose, partner drug, and follow-up limit attribution
Pragman et al., 2024 [39]	Longitudinal observational case-control; longitudinal case-control (n=81); upper airway/sputum 16S	Frequent-exacerbator phenotype	Lower sputum diversity and greater inflammation	Upper-airway contribution and treatment confounding
Goolam Mahomed et al., 2021 [38]	Cross-sectional geographically specific cohort; South African stable/exacerbated COPD; sputum/targeted metagenomics	Stable vs exacerbation	Population-specific bacterial and viral observations	Small sample; pooled virome sequencing; replication required
Lin et al., 2025 [30]	Discovery/validation cross-sectional multi-omic; discovery/validation cohorts; BAL metagenomics, transcriptomics, qPCR	COPD severity strata	Microbial-immune and resistance-gene associations	Cross-sectional; severity and chronic treatment pressure may confound
Hu et al., 2025 [26]	Small paired pre-/post-treatment observational; paired AECOPD samples (n=24); stool/pharyngeal 16S and metabolites	Before vs after combined treatment	Gut and pharyngeal changes after treatment	No untreated comparator; small sample; drug-specific effects unresolved
Liu et al., 2024 [28]	Cross-sectional virome case-control/classifier development; case-control (n=92); fecal virus-like particle metagenomics/16S	COPD vs controls	Gut virome and phage-bacterial differences; internal classifier	Single-region cross-sectional study; incomplete databases; no external validation
Moon et al., 2026 [19]	Prospective longitudinal observational cohort; prospective cohort; COPD (n=43); controls (n=10); 129 annual sputum samples; 16S	Stable COPD; two-year follow-up; ICS, smoking, lung function	Clinical factors associated with distinct longitudinal taxonomic trajectories	Small all-male Korean cohort; annual intervals; sputum admixture; residual time-varying confounding
Liu et al., 2026 [22]	Hospital observational cohort with small prospective subcohort; hospital cohort (n=202); prospective sputum subcohort (n=30); days 1 and 5; 16S	Eosinophilic vs non-eosinophilic AECOPD during treatment	Short-term clinical outcomes and microbiome changes differed by eosinophil phenotype	Very small microbiome subcohort; treatment components not isolated; observational comparison
van Iersel et al., 2025 [35]	Exploratory randomized intervention with baseline case-control comparison; age-matched baseline comparison (n=64); randomized exploratory intervention (n=32); fecal 16S	Stable COPD; multi-nutrient supplement containing prebiotic fibers for three months	Baseline differences observed; no significant intervention effect on microbiome or systemic inflammation	Only 16 per arm; multi-component product prevents attribution to prebiotic fibers alone

Methodological heterogeneity as a central explanation for inconsistent findings

Sampling, Low Biomass, and Contamination

Methodological heterogeneity is a central interpretive theme of this review, not a peripheral technical limitation. Differences in sampling site, specimen quality, microbial biomass, contamination prevention, laboratory processing, sequencing depth and platform, bioinformatic workflow, statistical analysis, population composition, clinical state, and medication exposure can materially alter apparent microbial profiles. Methodological and clinical noncomparability is therefore a principal explanation for disagreement among COPD microbiome studies and may be mistaken for genuine biological inconsistency. Sputum is accessible but vulnerable to oral admixture, whereas BAL more closely approximates the lower airway but is invasive and selects smaller cohorts. Respiratory specimens are low biomass, so reagent, extraction-kit, environmental, operator, and cross-sample contaminants can rival or exceed the biological signal. Contamination is an inherent continuum of risk in collection and processing, not a problem that exists only when negative extraction controls, blank sequencing controls, or contaminant-removal procedures are unreported. Even well-controlled studies may retain contaminants, while post hoc computational removal can also discard genuine low-abundance organisms. Consensus recommendations therefore emphasize aseptic collection, randomized and batch-aware processing, field and procedural controls where relevant, multiple negative controls, positive controls or mock communities, quantitative biomass measurement, transparent prevalence- and frequency-based contaminant identification, and reporting of both control results and sensitivity analyses [36,37]. Controls characterize and reduce uncertainty; they do not provide a binary guarantee that all retained taxa are biological.

Biomass, Sequencing, and Functional Inference

Analytical choices materially change apparent profiles. 16S rRNA sequencing is accessible, but extraction method, primer and target region, copy-number variation, and limited species or strain resolution affect results. Shotgun metagenomics can improve taxonomic, strain, gene, and pathway resolution, yet it has major limitations in low-biomass respiratory samples. Abundant human DNA can dominate libraries, reduce effective microbial depth, increase cost, and motivate host-depletion procedures that may introduce differential loss or bias. Greater nominal read count does not ensure adequate effective microbial sequencing depth, and rare organisms or resistance genes may remain below detection. Incomplete and uneven reference databases impair classification, particularly for fungi, viruses, phages, and poorly characterized strains. DNA sequencing cannot distinguish viable or metabolically active organisms from extracellular DNA or remnants of dead cells, and detection of a gene does not establish its expression, transferability, or phenotypic effect. Computational pathway reconstruction therefore represents predicted functional potential and is vulnerable to annotation error and overinterpretation. Extraction batch, index hopping, cross-sample contamination, host read removal, assembler and classifier choice, and filtering thresholds can further change results [36,37].

Relative abundance is compositional and cannot determine absolute microbial burden: a taxon may appear to increase when other organisms decline even if its quantity is unchanged. Total bacterial and fungal burden should therefore be measured when feasible using qPCR, digital PCR, flow cytometry, or another validated method. Known-concentration spike-ins can support the estimation of extraction recovery and technical variation, but they do not automatically provide comparable cell counts. Quantitative estimates remain sensitive to extraction efficiency, differential cell lysis, matrix effects, assay detection limits, amplification efficiency, variation in 16S or fungal-marker copy number per genome, the distinction between DNA and viable cells, and calibration across laboratories. Spike-in identity, concentration, recovery, normalization, and uncertainty should be reported. Sequencing depth should be justified because nominal read count may not represent microbial depth in host-DNA-rich specimens. Studies should state biomass, detection limits, extraction efficiency, total load, microbial and host read counts, normalization, and whether conclusions use relative or absolute abundance. DNA-based pathway reconstruction represents functional potential; activity requires more direct transcriptomic, proteomic, metabolomic, culture-based, or validated functional evidence.

Cross-Cohort Reproducibility

Ecological and taxonomic reproducibility should be distinguished explicitly. Ecological reproducibility refers to recurrence of higher-order properties, such as reduced diversity, pathobiont-dominated states, altered interaction networks, instability, or changed total microbial burden. In this review, an ecological feature was considered potentially reproducible only when it recurred in at least two independent cohorts or in a multicohort analysis, showed a comparable direction in a similar clinical state, and was not wholly dependent on one analytical pipeline; concordant longitudinal evidence strengthened this designation. This pragmatic definition is not a validated threshold and does not imply causality. Taxonomic reproducibility requires the same organisms, in comparable directions and effect sizes, across cohorts. COPD studies show greater convergence for ecological states than for individual taxa or classifiers. Population composition, climate, diet, ancestry, smoking, healthcare exposure, specimen handling, biomass, sequencing depth, pipeline, database version, filtering, and statistical model can all alter taxonomic results. The South African cohort illustrates geographic dependence [38], whereas frequent-exacerbator and SPIROMICS studies show partially convergent ecological associations despite different sampling and populations [14,15,39]. No taxonomic classifier is sufficiently stable across settings for routine use.

Confounding beyond COPD: host, behavioral, environmental, clinical, and treatment factors

**Figure 2:**
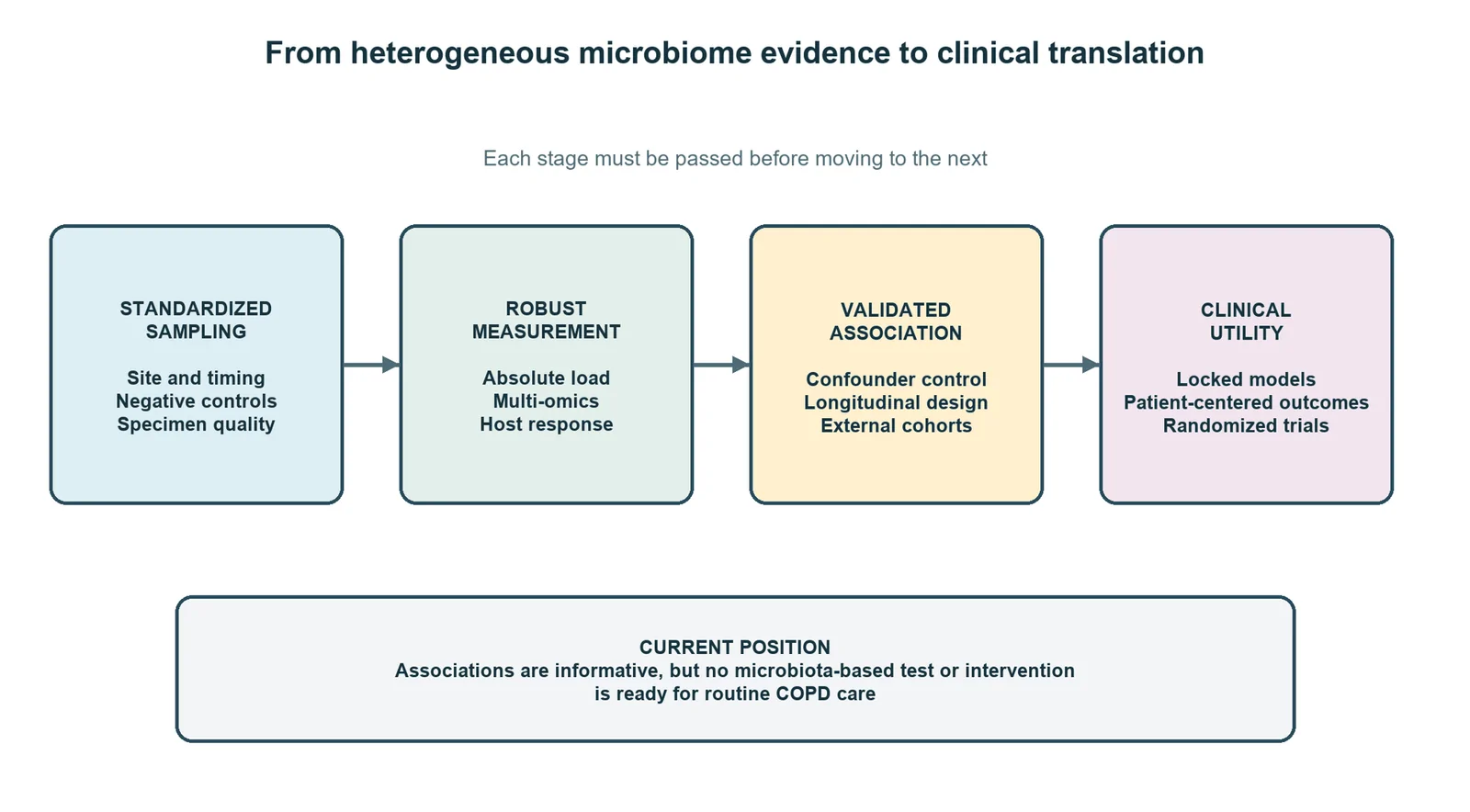
Translational pathway for COPD microbiome research. Standardized sampling and robust measurement must precede validated longitudinal associations, external reproducibility, and trials demonstrating patient-centered clinical utility. COPD: chronic obstructive pulmonary disease

The microbiome is influenced by many factors other than COPD, and these variables can differ systematically between patients and controls. Important demographic and social confounders include age, sex, ancestry, socioeconomic status, and geography. Host-related factors include body mass index, diet, oral health, dentition, immune phenotype, bowel function, and comorbidities. Behavioral and environmental factors include smoking status, pack-years, electronic-cigarette exposure, alcohol use, physical activity, occupation, pollution, household environment, and recent travel. Clinical factors include COPD severity, exacerbation history, infection, hospitalization, oxygen therapy, and whether sampling occurred during stability, exacerbation onset, treatment, or recovery. Treatment-related factors include antibiotics, ICS, systemic corticosteroids, proton-pump inhibitors, probiotics, and other drugs. Investigators should report medication class, formulation, dose, duration, cumulative exposure, adherence, indication, and timing relative to sampling. Statistical adjustment reduces but does not eliminate residual confounding, and confounding by indication may make a treatment-associated profile appear disease-associated [7,8,13-15,29,32,38,39]. Figure 2 presents the sequential evidentiary requirements for translation from heterogeneous observational findings to clinical utility.

Clinical interpretation and translational implications

GOLD 2026 recognizes associations between lower-airway dysbiosis, COPD characteristics, and exacerbations but emphasizes that longitudinal and interventional evidence is insufficient to establish direction or clinical utility [1]. Across recent studies, lower diversity, pathobiont-predominant states, and altered interaction networks recur in selected cohorts, yet their magnitude and taxonomic composition vary. Research sequencing therefore does not replace clinical assessment, culture, or susceptibility testing, and no microbiota-derived test or intervention is part of routine COPD care [1,5-8,14-21,34,35].

Antibiotics are not indicated routinely for every exacerbation, and sputum purulence is not equivalent to microbiologically confirmed bacterial infection. Purulence can increase the clinical probability of bacterial involvement, but it neither identifies a pathogen nor replaces culture, molecular testing when indicated, susceptibility testing, or assessment of alternative causes. Clinical features, prior microbiology, local resistance patterns, exacerbation severity, and the need for mechanical ventilation inform treatment [1,40]. For outpatient COPD exacerbations when antibiotics are indicated, GOLD 2026 recommends a course of no more than five days. In other treatment settings, GOLD describes a usual total duration of five to seven days, individualized according to clinical response, pathogen risk, complications, and local antimicrobial guidance [1]. These setting-specific recommendations should not be read as consecutive courses. In microbiome studies, antibiotic indication, class, route, start and stop dates, duration, and timing relative to sampling should be treated as direct ecological exposures and potential confounders.

Airway microbiota remains an investigational source of phenotypic and prognostic biomarkers, not a treatment-selection tool. Relative abundance does not establish microbial burden, infection, causal activity, or drug responsiveness. Clinical translation requires analytical validity, temporal stability, external reproducibility, calibration, incremental value beyond exacerbation history, symptoms, spirometry, blood eosinophils, and conventional microbiology, and ultimately evidence that biomarker-guided care improves patient outcomes [7,8,14,15,23].

Gut microbiome research must separate biomarker development from causal biology. Classifiers require locked models, external validation, calibration, clinical-utility analysis, and comparison with established predictors; causal claims require temporal ordering, appropriate comparators, confounder control, mediation or triangulation, and preferably intervention. Current human evidence cannot distinguish whether gut dysbiosis is a cause, consequence, treatment effect, shared marker, or combination of these processes. Repeated pre-treatment and post-treatment sampling and explicit causal designs are needed [9-13,28].

Microbiome change is not a validated surrogate for clinical benefit in COPD. Trials should prespecify one primary patient-centered outcome, an appropriate follow-up interval, and a minimal clinically important difference where available. In stable frequent exacerbators, annualized moderate-to-severe exacerbation rate or time to next exacerbation is usually the most directly relevant primary endpoint. In hospitalized AECOPD, treatment failure, length of stay, need for ventilatory support, readmission, and short-term mortality are more appropriate. Longer disease-modification studies should prioritize health-related quality of life, lung-function decline, hospitalization, and survival. Composition, diversity, load, metabolites, host-response measures, and resistome changes should remain secondary or mechanistic unless formally validated as surrogates. Blood eosinophil count is established for selected COPD decisions; broader references to inflammatory markers should not imply routine utility for C-reactive protein, cytokines, or experimental measures [8,13-15,23,29].

Limitations

This review integrates airway and gut evidence within one clinical framework and distinguishes exploratory findings from guideline-supported practice. Its focused narrative design permits a broad contemporary synthesis but is not comprehensive and does not provide pooled estimates, formal certainty grading, or validated study-level risk-of-bias ratings. The absence of formal risk-of-bias assessment means that differences in selection bias, confounding, missing data, outcome measurement, selective reporting, and multiplicity were appraised narratively rather than scored systematically; this limits comparisons of evidential credibility across studies. PubMed/MEDLINE was the only systematically searched bibliographic database; Embase, Scopus, Web of Science, CINAHL, preprint servers, and grey literature were not systematically searched. The English-language restriction may be especially problematic in a geographically heterogeneous field because it can omit relevant regional cohorts and introduce language bias. Exact search-result, deduplication, full-text exclusion, and inclusion counts were not prospectively archived. Screening, selection, data charting, and qualitative appraisal were performed by one author without duplicate review or a prespecified protocol, and citation tracking involved judgment. These limitations materially increase susceptibility to selection, confirmation, and interpretation bias. The review methodology is therefore not fully reproducible or exhaustive. Seminal pre-2021 studies were used only for historical and methodological context, not as part of the prespecified recent-study window. Conclusions should be interpreted in light of these review-level limitations as well as clinical, sampling, and analytical heterogeneity in the underlying studies. Table 1 is a purposively selected representative subset rather than an exhaustive study inventory, and the manuscript does not provide a study-by-study matrix for negative controls, positive controls, spike-ins, biomass measurement, effective microbial sequencing depth, batch handling, or absolute versus relative abundance. These omissions reduce auditability and prevent a systematic comparison of methodological quality across the entire evidence base.

Recommendations and future research

Priority 1: Minimum Methodological and Reporting Standards

Current clinical decisions should follow validated COPD guidelines, conventional microbiology, exacerbation history, symptoms, spirometry, and established biomarkers such as blood eosinophil count when indicated. Microbiome findings should not be used to select antibiotics, ICS, probiotics, or FMT outside research protocols. Future studies should report major host and treatment confounders, clinical state and sample timing, negative and positive controls, biomass, effective microbial sequencing depth, extraction and analytical batch effects, and the complete bioinformatic workflow; absolute load should accompany relative abundance whenever feasible [1,14,15,29].

Priority 2: Longitudinal Validation and Causal Investigation

Multicenter cohorts should use repeated paired airway and fecal sampling; integrated bacteriome, mycobiome, virome, resistome, metabolome, and host-response measurements; prespecified analyses; diverse external validation; and explicit assessment of time-varying treatment exposure. Mechanistic studies should distinguish measured activity from predicted functional potential and causal effects from markers of treatment exposure or advanced disease. Shared reference materials, negative and spike-in controls, absolute quantification, and interoperable standards are essential for cross-laboratory reproducibility [8,13,14,21,23,26,29].

Priority 3: Clinical Intervention and Utility

Adequately powered randomized trials should define one patient-centered primary outcome, an appropriate follow-up interval, safety outcomes, and a prespecified analysis plan. Microbiome, metabolite, inflammatory, and resistome changes should remain secondary or mechanistic outcomes unless formally validated as surrogates. Biomarker studies require locked models, calibration, external validation, assessment of incremental value beyond established predictors, and evidence that biomarker-guided care improves outcomes before clinical implementation.

## Conclusions

Airway and gut microbial features are associated with COPD phenotypes and outcomes, but the underlying evidence bases are unequal. Airway evidence includes larger phenotyped cohorts, repeated sampling, bronchoscopic studies, and limited randomized treatment comparisons, whereas gut-lung evidence remains predominantly cross-sectional, indirect, and dependent on biological plausibility; microbiota-directed intervention evidence is the least mature and does not support routine clinical use. Recurrent airway ecological patterns do not constitute a disease-specific taxonomic signature, and relative abundance without load quantification may mislead. The gut-lung axis is plausible but not causally established in humans. Three priorities should guide the field: standardized sampling and absolute microbial-load measurement; longitudinal, paired airway-gut designs capable of testing temporal and causal hypotheses; and externally validated intervention trials with a prespecified patient-centered primary outcome.
